# Stronger together: community participation, structural stigma, and depression among sexual and gender minority adults in 28 European countries

**DOI:** 10.1007/s00127-022-02385-w

**Published:** 2022-11-24

**Authors:** Banu C. Ünsal, Zsolt Demetrovics, Melinda Reinhardt

**Affiliations:** 1 Doctoral School of Psychology, ELTE Eötvös Loránd University, Budapest, Hungary; 2 Institute of Psychology, ELTE Eötvös Loránd University, Budapest, Hungary; 3 Centre of Excellence in Responsible Gaming, University of Gibraltar, Gibraltar, Gibraltar

**Keywords:** Community participation, Structural stigma, Depression, Minority stress, Intersectionality, Sexual and gender minorities

## Abstract

**Purpose:**

Although discriminatory laws, policies, and public attitudes (i.e., structural stigma) are linked to adverse mental health outcomes among sexual and gender minority (SGM) populations, little attention has been paid to protective factors, such as community participation, about which inconsistencies exist whether it ameliorates or exacerbates mental health burdens. Thus, we examined the mediator roles of identity disclosure and victimization and the moderator role of structural stigma in the association of community participation with depression.

**Methods:**

Data from the EU-LGBTI-II survey assessing community participation, identity disclosure, victimization, and depression among sexual minority men (*n* = 62,939), women (*n* = 38,976), and gender minority adults (*n* = 15,845) in 28 European countries were used. Structural stigma was measured as discriminatory legislation, policies, and societal attitudes using publicly available data.

**Results:**

Findings showed that community participation predicted lower and higher levels of depression through identity disclosure and victimization, respectively. For sexual minority men and women, structural stigma moderated the indirect effect through identity disclosure, with a larger effect in higher structural stigma countries. Only for sexual minority men, the indirect effect through victimization was also moderated, with a larger effect in high-stigma countries. For gender minorities, no moderation effect was found.

**Conclusions:**

Community participation is differentially linked to depression through identity disclosure and victimization, and as a function of structural stigma. It can be a double-edged sword, especially for sexual minority men in high-stigma countries, who are expected to pay the price while enjoying its benefits, highlighting the targets and considerations for interventions.

## Introduction

Sexual (i.e., whose sexual identity, attraction, or behavior is not exclusively heterosexual) and gender (i.e., whose sex assigned at birth does not align with their current gender identity or expression) minority (SGM) individuals have consistently been found to experience more symptoms of mental health problems including depression as compared to their cisgender and heterosexual counterparts [1, 2]. These disproportionately higher rates of depression among SGM populations are suggested to result from increased exposure to stigma and associated minority stress [3–5].

Stigma compromises the mental health of SGM individuals at multiple levels, which are structural, interpersonal, and individual, respectively [4]. Structural stigma is defined as discriminatory state laws, social policies, and public attitudes that restrict SGM individuals’ opportunities or fail to protect their fundamental human rights (e.g., same-sex marriage bans, not legally recognizing trans identities [6]). Interpersonal stigma manifests in social interactions as prejudice-based events, such as victimization [3, 4]. Finally, individual stigma refers to alterations in the stigmatized individuals’ cognitive, emotional, or behavioral processes in response to the stigma, which comprises maladaptive coping strategies, such as concealment of identity [3, 4]. So far, previous studies have indicated that structural stigma impairs the psychological well-being of SGM individuals directly [7, 8] and indirectly by synergistically interacting with stigma at other levels [9–12].

Despite the increasing interest in structural stigma, up to now, little attention has been paid to protective factors. Community-level variables such as support from, feeling connected to, or participating in SGM communities have been considered protective factors against adverse impacts of stigma on mental health [3, 13]. Although there is a degree of uncertainty around the terminology of community-level factors (e.g., connectedness, involvement, participation, engagement [13, 14]), community participation can be broadly defined as behaviorally participating in a community through professional groups or recreational activities to build reciprocal relationships with that community [15]. It is suggested to play an ameliorative role by allowing individuals to receive social support from similar others, engage in altruistic behaviors, and meet the need for belongingness [3, 13, 16].

However, previous studies reported conflicting results regarding the impact of community participation on mental health, from positive to negative, indicating that it is not always beneficial [14, 17–21]. These inconsistencies might be explained by individual and interpersonal stigma. As previously suggested, community participation is associated with decreased concealment and increased disclosure of identity via providing individuals with supportive social networks in which their sense of acceptance is fostered [22, 23]. Similarly, developmental theories of coming out assert that community participation coincides with the coming out process and further encourages individuals to disclose to more people, because, among SGM communities and advocacy groups, coming out is considered politically and socio-economically beneficial for the whole community [24]. Thus, one of the mechanisms through which community participation is associated with *better* mental health outcomes might be increased identity disclosure. On the other hand, because community participation requires being publicly open about one’s identity to some extent and increases one’s exposure as a minority, [25, 26] another mechanism through which community participation is associated with *adverse* mental health outcomes might be increased exposure to interpersonal stigma, such as victimization.

Furthermore, the extent to which these mediating mechanisms operate might depend on structural stigma. Because the presence of SGM communities in a given locale differs as a function of its structural climate and restricting the legal establishment and operation of SGM organizations is itself a form of structural stigma [9, 12], individuals in less stigmatizing contexts have more opportunities to participate in the community [27]. It was previously reported that sexual minority men in neighborhoods with more representations of gay men were more likely to be involved in their community [28], suggesting that community participation might be more beneficial in low-stigma countries.

Nonetheless, some evidence suggests that community participation is most beneficial in more stigmatizing contexts [29, 30]. Due to higher rates of interpersonal and individual stigma experienced by SGM individuals in high-stigma contexts [12], these individuals’ need to participate in and potential to benefit from the community might be greater. In other words, community participation might improve mental health through increased identity disclosure, which may be augmented in high-stigma countries, since individuals in such countries conceal their identity from more people [9, 10]. On the other hand, community participation might counterproductively impair mental health through increased exposure to victimization, which may also be amplified in high-stigma countries due to more frequent exposure to victimization in such countries [10, 11].

Considering the inconsistent empirical findings presented above, it is crucial to investigate the structural, interpersonal, and individual factors to understand better the differential impacts of community participation on mental health. Thus, this study aimed to examine the mediator roles of identity disclosure and victimization in the association of community participation with depression among SGM individuals living in 28 European countries and the moderator role of structural stigma in these associations. To the best of our knowledge, this is the first cross-national study that examines community participation and depression among SGM individuals. This study also investigates the contextual (i.e., structural stigma), interpersonal (i.e., victimization), and individual (i.e., identity disclosure) mechanisms of this association in a large European sample of SGM individuals.

**Fig. 1:**
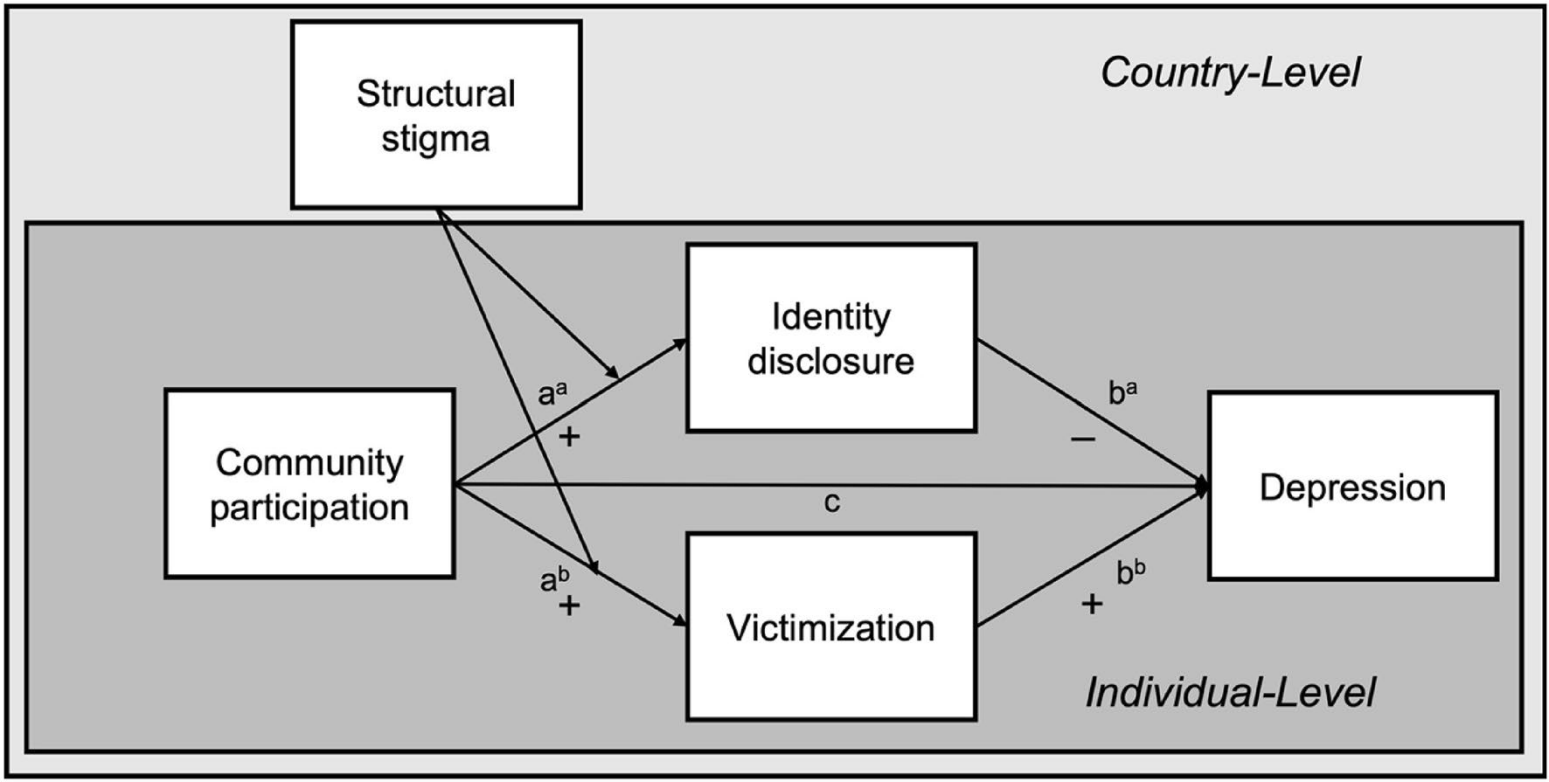
Proposed multilevel model of the association between community participation and depression among sexual and gender minority individuals across Europe, identity disclosure and victimization as individual-level mediators, and structural stigma as the country-level moderator

We hypothesized that identity disclosure and victimization would mediate the association of community participation with depression (see Fig. 1). Accordingly, we expected that community participation would be associated with increased identity disclosure (Fig. 1, path a^a^), which would predict *lower* levels of depression (Fig. 1, path b^a^). On the contrary, we also expected that community participation would predict increased exposure to victimization (Fig. 1, path a^b^), which would be associated with *higher* depressive symptoms (Fig. 1, path b^b^). We further hypothesized that structural stigma would moderate the associations of community participation with identity disclosure and victimization. However, given the previous contradictory findings and scarcity of research, we did not specify hypotheses about how results would differ between countries. In addition, because subgroups of SGM populations (i.e., sexual minority men, sexual minority women, and gender minority adults) demonstrate variability in community participation [13] and are affected by distinct forms of structural stigma [27, 31], by adopting an intersectional approach [5, 32], the proposed model was examined separately for these groups.

## Methods

### Sample

We used data from the second European Union Lesbian, Gay, Bisexual, Transgender, and Intersex (EU-LGBTI-II) survey [33]. In 2019, the EU Agency for Fundamental Human Rights conducted a large-scale survey to assess LGBTI individuals’ experiences in 30 European countries. 139,799 LGBTI respondents aged 15 or older and living in 28 EU member states and two candidate states (i.e., Serbia and North Macedonia) completed the survey. However, due to significant differences between experiences of SGM youth and adults [4] and between structural climates of the EU member states and candidate states, we only used data from participants aged 18 or older, living in 28 EU member states, and self-identified as sexual (e.g., lesbian, gay, bisexual) or gender (e.g., trans, non-binary, gender-fluid) minority. Thus, the sample consisted of 117,760 adults, 62,939 (53.4%) identified as sexual minority men, 38,976 (33.1%) as sexual minority women, and 15,845 (13.5%) as gender minority. The details of the survey development have been explained elsewhere [34].

### Individual-level measures

#### Sexual and gender identity

Gender identity was assessed with two questions. First, participants answered a question about their current gender identity, with response options including “woman/girl,” “man/boy,” “trans woman/girl,” “trans man/boy,” “non-binary or genderqueer or agender or polygender or gender-fluid,” and “other.” Participants who selected the trans or non-binary options above were categorized as gender minorities. Respondents who selected the remaining options answered another question asking if they are/*were* trans persons to differentiate those with a trans history but without a current trans identity. Individuals responding yes to this question were also categorized as gender minorities. Sexual identity was assessed with the question asking participants to select the option that best matches their sexual orientation, including “lesbian,” “gay,” “bisexual,” “heterosexual/straight,” and “other.” Individuals who identified as heterosexual/straight but were not previously categorized as gender minority were not allowed to continue taking the survey. The remaining respondents who were not previously included in the gender minority category were categorized as sexual minority men or women based on their gender identity, resulting in three mutually exclusive subsamples [34].

#### Community participation

Community participation was measured by asking participants if they were involved in one or more organizations for SGM people. Respondents selected all the following items that applied to them: “I am an active member or volunteer,” “I am in a regular contact,” “I follow their activities,” and “I am a financial supporter.” Selected items were coded as 1, whereas not selected items were coded as 0. Each participant's total community participation score was calculated by averaging the scores across the four items above.

#### Victimization

Victimization was assessed as the frequency of physical or sexual attacks in the last 5 years due to sexual or gender identity. Response options included: “never [coded = 0],” “once [coded = 1],” “twice [coded = 2],” “3–5 times [coded = 4],” “6–10 times [coded = 8],” “more than 10 times [coded = 11],” and “all the time [coded = 15].” Based on the skewed distribution of the variable, the scores were dichotomized to include experiences of *no victimization* [coded = 0] and *at least one victimization* [coded = 1].

#### Identity disclosure

Identity disclosure was assessed by asking participants how many people they are open to about their sexual or gender identities among the following groups: “family members other than their partner(s),” “friends,” “neighbors,” “work colleagues,” “schoolmates/university co-students,” “immediate superior/head of department,” “customers, clients, etc. at work,” and “medical staff/healthcare providers.” Response options included the following: “none [coded = 0],” “a few [coded = 1],” “most [coded = 2],” “all [coded = 3],” and “doesn’t apply to me.” An average identity disclosure score was calculated by summing up the scores obtained from the eight groups of people above divided by the number of groups applying to the participant.

#### Depression

Depression was measured with the question: “Have you been feeling downhearted or depressed over the last 2 weeks?” Response options involved: “at no time [coded = 0],” “some of the time [coded = 1],” “less than half of the time [coded = 2],” “more than half of the time [coded = 3],” “most of the time [coded = 4],” and “all the time [coded = 5].” Similar single-item measures of depression were found to be in good agreement with and perform as well as multiple-item measures [35].

#### Covariates

Because age cohort [36], ethnic minority status [3, 13], and socioeconomic status [37] create additional privilege or oppression dynamics affecting individuals’ access to interpersonal and structural resources, following previous research on structural stigma [10, 11], age, ethnic minority status, and income level were included as individual-level covariates.

### Country-level measures

#### Structural stigma

Following previous research on structural stigma [9, 10, 12], we created two measures (i.e., sexual orientation- and gender identity-related measures) of structural stigma in 2019 for each country. First, two indices of laws and policies regarding sexual orientation and gender identity were created using the 2019 Rainbow Index of International Lesbian, Gay, Bisexual, Trans, and Intersex Association in Europe [38]. For the sexual orientation index, countries were given a positive point for national/federal applications and a half-point for only regional applications of their sexual orientation-related protective laws and policies across five domains (i.e., equality and non-discrimination, family, hate crime and hate speech, civil society space, and asylum). The scores were then summed and standardized. We followed the same procedure for the gender identity index by only including gender identity-related laws and policies from the above domains. This index also had one domain concerning only gender minorities (i.e., legal gender recognition and bodily integrity).

These indices were then combined with a measure of attitudes toward SGM individuals from the European Commission’s “Special Eurobarometer 493: Discrimination in the EU (including LGBTI),” which included data from citizens of 28 EU member states in 2019 [39]. The attitudes toward SGM individuals were measured with a question asking people on a 10-point scale to rate how comfortable they would feel about having “a gay, lesbian or bisexual person” (i.e., attitudes toward sexual minorities) and “a transgender person” (i.e., attitudes toward gender minorities) in the highest elected political position in their country, with higher scores representing more positive attitudes. We standardized the mean scores of attitudes for each country. These standardized measures of public attitudes and the standardized indices of laws and policies above were averaged and then inversed to ease the interpretation of structural stigma, with higher scores representing higher levels of structural stigma. The final sexual orientation structural stigma scores ranged between − 1.45 in the UK and 1.71 in Bulgaria. The final gender identity structural stigma scores varied between − 1.34 in Malta and 1.63 in Lithuania.

#### Covariates

Since general inequalities (e.g., income inequality) between the countries have been shown to be associated with depression [30, 37, 40], the gross domestic product (GDP) at purchasing power parity per capita in 2019 derived from the World Bank [41], Democracy Index [42], and Human Development Index in 2019 [43] were included as country-level covariates.

### Statistical analyses

Due to the nested structure of the data, multilevel mediation and moderated mediation analyses were conducted. Individual-level variables (i.e., community participation, identity disclosure, victimization, depression, and sociodemographic covariates) were included at level 1, and country-level variables (i.e., structural stigma, GDP, democracy index, and human development index) were modeled at level 2. The amount of missing data ranged from 0% (e.g., depression, identity disclosure, age) to 0.3% (e.g., income, victimization). Only participants with complete data on all study variables were included in the analyses. Maximum likelihood parameter estimations were used to estimate the fixed and random effects. MLmed Macro of SPSS [44] was used for analyses, allowing us to decompose the predictors into their within- and between-level components, calculate the within- and between-level indirect effects, test the index of moderated mediation, and automatically generate Monte Carlo Confidence Intervals (MCCI).

We started our analyses with fixed-effect models that included level-1 variables. We then (1) added level-1 random slopes (i.e., paths from the predictor to mediators) and intercepts, and (2) included the level-2 moderator and covariates in separate models. We conducted likelihood-ratio tests to examine each model’s goodness of fit [45]. Within-level effects obtained from the models run separately for the three subsamples were reported. Since symmetric confidence intervals (CI) are not applicable in multilevel models with unknown parameter sampling distributions [44], 95% MCCI based on 10,000 Monte Carlo samples were reported. The conditional indirect effects were considered significant if the MCCI did not include zero. Interactions were probed for one standard deviation below and above the means of structural stigma scores to represent the indirect effects conditioned on high vs. low structural stigma.

## Results

### Descriptive statistics

More than half of the sample were younger than 30 years of age (*n* = 68,922, 58.5%) and university graduates (*n* = 61,332, 52.1%). Most of the participants were living in an urban area (*n* = 104,548, 88.8%) and in a relationship (*n* = 84,526, 71.8%). The highest number of participants contributing to subsamples of sexual minority men (*n* = 8,219, 13.1%) and sexual minority women (*n* = 6,772, 17.4%) lived in Spain, and gender minority adults (*n* = 2,561, 16.2%) lived in Germany. The lowest number of participants contributing to subsamples of sexual minority men (*n* = 195, 0.3%), women (*n* = 100, 0.3%), and gender minority adults (*n* = 28, 0.2%) lived in Luxembourg (Table 1).

**Table 1:** Sociodemographic characteristics of subsamples and the total sample of the EU-LGBTI-II Survey

	*n* (%)	*n* (%)	*n* (%)	*n* (%) ^a^
	Sexual minority men (*n* = 62,939)	Sexual minority women (*n* = 38,976)	Gender minority adults (*n* = 15,845)	Total (*N* = 117,760)
*Sex assigned at birth*				
Male	62,939 (100)	0	5513(34.8)	68,452 (58.1)
Female	0	38,976 (100)	9491 (59.9)	48,467 (41.2)
Other	0	0	841 (5.3)	841 (0.7)
*Age*				
18–29	30,450 (48.4)	27,259 (69.9)	11,213 (70.8)	68,922 (58.5)
30–39	15,052 (24.0)	7027 (18.1)	2469 (15.6)	24,548 (20.8)
40–49	9799 (15.6)	3011 (7.7)	1166 (7.3)	13,976 (11.9)
50–59	5723 (9.1)	1235 (3.1)	698 (4.4)	7656 (6.5)
60 or older	1915 (3.1)	444 (1.1)	299 (1.8)	2658 (2.2)
*Sexual orientation*				
Gay	54,707 (86.9)	0	2053 (13)	56,760 (48.2)
Lesbian	0	19,576 (50.2)	2780 (17.5)	22,356 (19)
Bisexual	8232 (13.1)	19,400 (49.8)	5774 (36.4)	33,406 (28.4)
Heterosexual	0	0	1394 (8.8)	1394 (1.2)
Other	0	0	3779 (23.8)	3779 (3.2)
Don’t know	0	0	65 (0.4)	65 (0.1)
Ethnic minority status	5229 (8.3)	2456 (6.3)	1186 (7.5)	8871 (7.5)
*Income*				
Very low	2417 (3.8)	1494 (3.8)	1256 (7.9)	5167 (4.4)
Low	4837 (7.7)	3623 (9.3)	2091 (13.2)	10,551 (9)
Low-to-middle	13,332 (21.2)	9899 (25.4)	4311 (27.2)	27,542 (23.4)
Middle-to-high	17,877 (28.4)	11,681 (30.0)	4390 (27.7)	33,948 (28.8)
High	15,502 (24.6)	8980 (23.0)	2666 (16.8)	27,148 (23.1)
Very high	8860 (14.1)	3235 (8.3)	1087 (6.9)	13,182 (11.2)
*Education level*				
Less than a university degree	27,873 (44.3)	19,010 (48.8)	9545 (60.2)	56,428 (47.9)
University graduate	35,066 (55.7)	19,966 (51.2)	6300 (39.8)	61,332 (52.1)
*Place of residence*				
Urban	56,145 (89.2)	34,515 (88.6)	13,888 (87.6)	104,548 (88.8)
Rural	6790 (10.8)	4459 (11.4)	1948 (12.3)	13,197 (11.2)
*Relationship status*				
Single	17,669 (28.1)	11,989 (30.8)	3576 (22.6)	33,234 (28.2)
In a relationship	45,270 (71.9)	26,987 (69.2)	12,269 (77.4)	84,526 (71.8)

^a^Percentages may not equal 100 due to missing data

### Sexual minority men

#### Identity disclosure and victimization as mediators of the association between community participation and depression

Fixed-effects multilevel mediation analysis examining whether community participation is associated with depression indirectly through identity disclosure and victimization demonstrated that, as hypothesized, indirect effects of community participation on depression through identity disclosure (*β* =  – 0.1913; 95%MCCI [ – 0.2062,  – 0.1766]; *p* < 0.001) and victimization (*β* = 0.0770; 95%MCCI [0.0680, 0.0861]; *p* < 0.001) were significant (see Table 2). Including level-1 random slopes and intercepts in the second model showed that associations of community participation with identity disclosure (*β* = 0.0551, *p* < 0.01) and victimization (*β* = 0.0035, *p* < 0.05) significantly varied across countries, and the model had a better fit (*∆χ*^*2*^(4) = 2872.3, *p* < 0.001). The variance components of the indirect effects through identity disclosure and victimization were 0.0025 (*SD* = 0.0503) and 0.0008 (*SD* = 0.0276), respectively. Thus, although indirect effects through identity disclosure and victimization were significant in all countries, these effects were much smaller or larger in some countries.

**Table 2:** Results of multilevel mediation and moderated mediation models for three subsamples^ab^

	Sexual minority men (*n* = 62,762)			Sexual minority women (*n* = 38,835)			Gender minority adults (*n* = 15,739)		
	Model 1	Model 2	Model 3	Model 1	Model 2	Model 3	Model 1	Model 2	Model 3
	*β (SE)*	*β (SE)*	*β (SE)*	*β (SE)*	*β (SE)*	*β (SE)*	*β (SE)*	*β (SE)*	*β (SE)*
CP Disclosure	0.8918^***^ (0.065)	0.9863^***^ (0.053)	0.8291^***^ (0.061)	0.5421^***^ (0.021)	0.5883^***^ (0.039)	0.6905^***^ (0.058)	0.8486^***^ (0.030)	0.8825^***^ (0.054)	0.9187^***^ (0.103)
CP Victimization	0.1648^***^ (0.008)	0.1641^***^ (0.016)	0.1273^***^ (0.019)	0.1731^***^ (0.010)	0.1645^***^ (0.016)	0.1936^***^ (0.024)	0.2503^***^ (0.016)	0.2439^***^ (0.020)	0.2333^***^ (0.044)
Disclosure Dep	– 0.2145*** (0.007)			– 0.3746*** (0.011)			– 0.2475*** (0.017)		
Victimization Dep	0.4673*** (0.017)			0.3939*** (0.023)			0.4566*** (0.032)		
CP Dep	0.1306*** (0.033)			0.1450** (0.044)			– 0.1814** (0.066)		
Int Disclosure			0.1630^**^ (0.048)			0.0936^*^ (0.040)			0.0283 (0.069)
Index			– 0.0350^****^			– 0.0351^****^			– 0.0070
Int Victimization			0.0430^**^ (0.016)			0.0243 (0.016)			– 0.0072 (0.028)
Index			0.0201^****^			0.0096			– 0.0033
Model Fit		*∆χ*^*2*^* (df)* 2872.3 (4)^***^	*∆χ*^*2*^* (df)* 35.8 (12)^***^		*∆χ*^*2*^* (df)* 644.4 (4)	*∆χ*^*2*^* (df)* 24.8 (12)^*^		*∆χ*^*2*^* (df)* 211.6 (4)	*∆χ*^*2*^* (df)* 18.5 (12)

*CP *Community participation, *Dep *Depression, *Int* Community participation x Structural stigma interaction

^a^Within-level fixed effects adjusted for age, income level, and ethnic minority status were reported

^b^3rd models were adjusted for GDP, democracy index, and human development index as well

*p < 0.05, **p < 0.01, ***p < 0.001, ****Monte Carlo Confidence Intervals does not contain zero

#### Structural stigma as the moderator

**Fig. 2:**
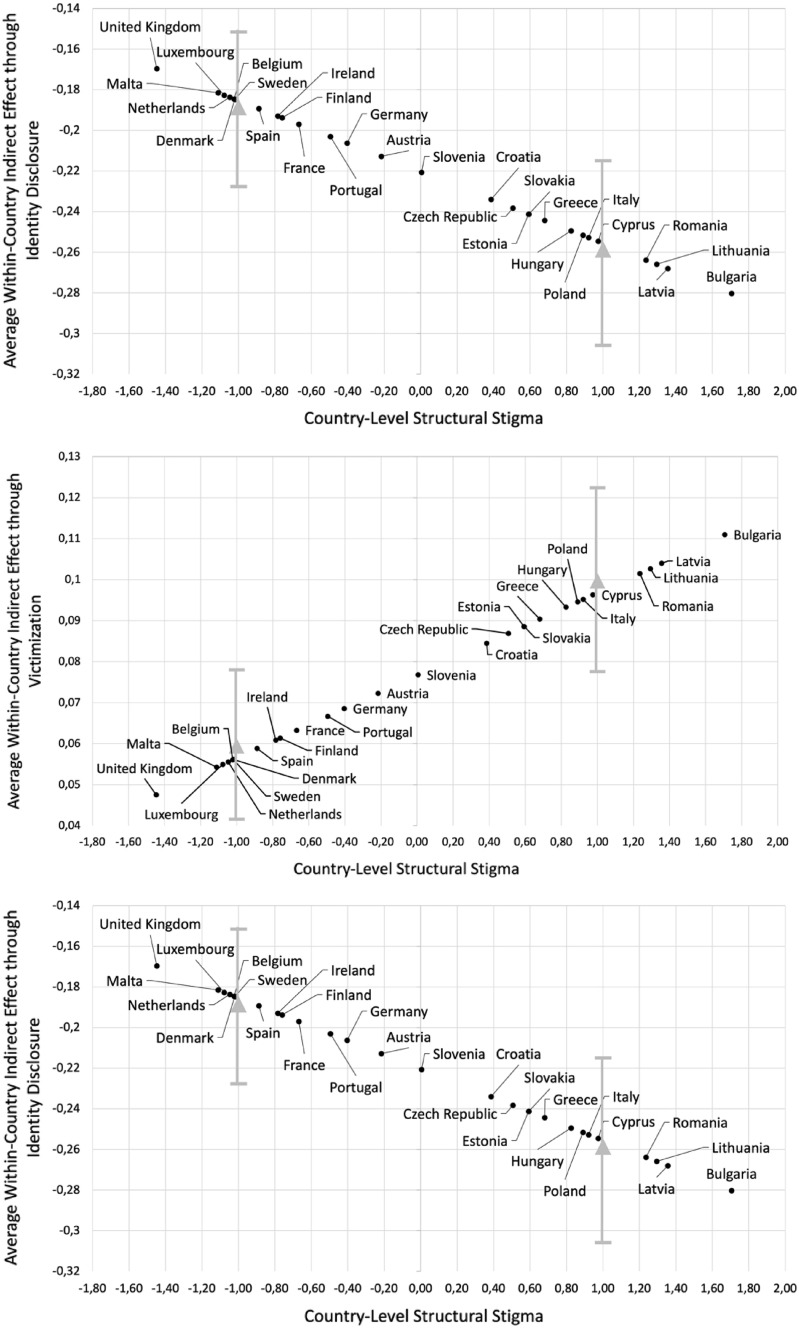
**a–c** Average within-country indirect effects of community participation on depression through (**a**) identity disclosure and (**b**) victimization among sexual minority men *(n* = 62,762)*,* and through (**c**) identity disclosure among sexual minority women (*n* = 38,835) as a function of 28 European countries’ structural stigma levels. Grey triangles represent effects conditioned on one standard deviation below and above the mean country-level structural stigma score, with bars representing 95% Monte Carlo Confidence Intervals

To account for these variances across countries, structural stigma regarding sexual orientation was added as the moderator in the third model, which had a better fit (*∆χ*^*2*^(12) = 35.8, *p* < 0.001) than previous model. Significant indices of moderated mediation were found for identity disclosure (*β* =  – 0.0350; 95%MCCI [ – 0.0557,  – 0.0145]) and victimization (*β* = 0.0201; 95%MCCI [0.0056, 0.0349]), indicating that, as hypothesized, structural stigma moderated the indirect effects. As illustrated in Fig. 2, the indirect effect of community participation on depression through identity disclosure was larger for high-stigma countries (*β* =  – 0.2477; 95%MCCI [ – 0.2825,  – 0.2159]; *p* < 0.001) as compared to low-stigma ones (*β* =  – 0.1778; 95%MCCI [ – 0.2066,  – 0.1505]; *p* < 0.001). A similar inversed pattern was found for victimization (see Fig. 2), such that the positive indirect effect of community participation on depression through victimization was greater in high-stigma countries (*β* = 0.0997; 95%MCCI [0.0776, 0.1224]; *p* < 0.001) compared to low-stigma ones (*β* = 0.0595; 95%MCCI [0.0416, 0.0780]; *p* < 0.001).

### Sexual minority women

#### Identity disclosure and victimization as mediators of the association between community participation and depression

Multilevel mediation model with fixed effects investigating the mediator roles of identity disclosure and victimization showed that, as hypothesized, indirect effects of community participation on depression via identity disclosure (*β* =  – 0.2030; 95%MCCI [ – 0.2222,  – 0.1842]; *p* < 0.001) and victimization (*β* = 0.0682; 95%MCCI [0.0575, 0.0793]; *p* < 0.001) were significant (see Table 2). Although including level-1 random slopes and intercepts in the second model did not result in a better model fit (∆*χ*^*2*^(4) = 644.4, *p* > 0.05), results showed that the association of community participation with identity disclosure significantly varied across countries (*β* = 0.0214, *p* < 0.05) with a variance of 0.0030 (*SD* = 0.0548). Thus, although community participation predicted lower levels of depression through identity disclosure, this effect was much smaller or larger in some countries. However, the association of community participation with victimization (*β* = 0.0028, *p* = 0.18) did not significantly vary across countries.

#### Structural stigma as the moderator

To test whether structural stigma could explain the cross-country variation, after including structural stigma regarding sexual orientation in the third model, which had a better fit (*∆χ*^*2*^(12) = 24.8, *p* < 0.05), confirming our hypothesis, a significant index of moderated mediation (*β* =  – 0.0351; 95%MCCI [ – 0.0652,  – 0.0051]) was found, indicating that structural stigma moderated the indirect effect through identity disclosure. Figure 2 shows that the indirect effect was larger in high-stigma countries (*β* =  – 0.2586; 95%MCCI [ – 0.3058,  – 0.2149]; *p* < 0.001) compared to low-stigma ones (*β* =  – 0.1885; 95%MCCI [ – 0.2277,  – 0.1514]; *p* < 0.001). Regarding victimization, contrary to hypotheses, a nonsignificant index of moderated mediation (*β* = 0.0096; 95%MCCI [ – 0.0032, 0.0226]) was found, confirming nonsignificant variation across countries detected in the previous model.

### Gender minority adults

#### Identity disclosure and victimization as mediators of the association between community participation and depression

**Fig. 3:**
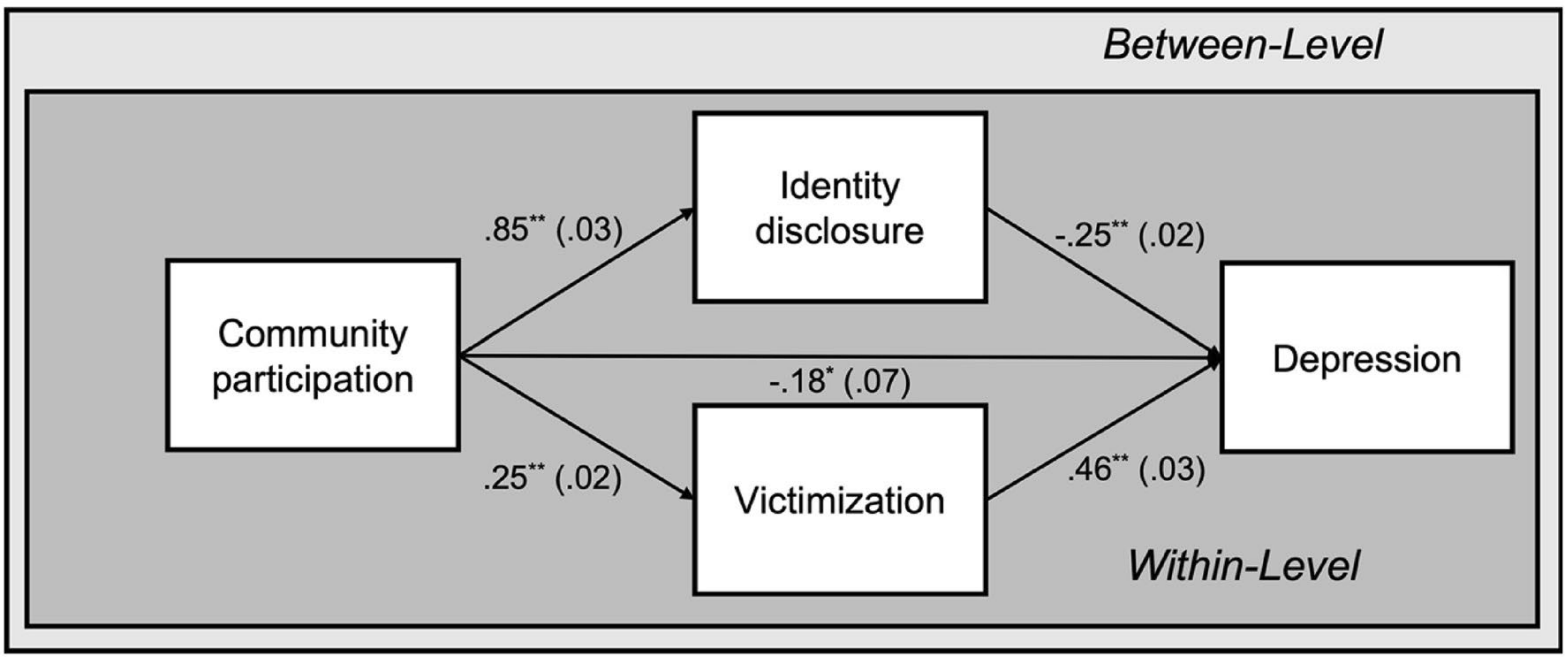
Multilevel mediation model of the association between community participation and depression among gender minority adults (*n* = 15,739) across Europe mediated by identity disclosure and victimization. Within-level fixed-effect estimates adjusted for age, ethnic minority status, and income level with standard errors in parentheses were reported. ^*^p < 0.01 ^**^p < 0.001

Fixed-effects multilevel mediation model testing whether identity disclosure and victimization mediate the community participation-depression association revealed that, as hypothesized, indirect effects through identity disclosure (*β* =  – 0.2101; 95%MCCI [ – 0.2426,  – 0.1788]; *p* < 0.001) and victimization (*β* = 0.1143; 95%MCCI [0.0932, 0.1364]; *p* < 0.001) were significant (see Fig. 3). However, neither the inclusion of level-1 random slopes and intercepts resulted in a significant model fit (*∆χ*^*2*^(4) = 211.6, *p* > 0.05) nor were there significant variances across countries regarding the association of community participation with identity disclosure (*β* = 0.0324, *p* = 0.07) and victimization (*β* = 0.0016, *p* = 0.56). Thus, for gender minority adults, contrary to our hypotheses, indirect effects did not significantly vary across countries. Results of the third model confirmed this with nonsignificant indices of moderated mediation for identity disclosure (*β* =  – 0.0070; 95%MCCI [ – 0.0414, 0.0276]) and victimization (*β* =  – 0.0033; 95%MCCI [ – 0.0288, 0.0223]), and a nonsignificant model fit (*∆χ*^*2*^(12) = 18.5, *p* = 0.10).

## Discussion

Because previous literature reported contradictory results regarding the role of community participation in SGM individuals’ mental health [14, 19–21, 46], using a large sample of SGM adults living in 28 European countries, we attempted to explain the mechanisms of this differential impact of community participation on depression by including individual (i.e., identity disclosure), interpersonal (i.e., victimization), and contextual (i.e., structural stigma) factors. Our results demonstrated that identity disclosure and victimization mediated the community participation–depression association for sexual minority men, women, and gender minority adults. In other words, community participation predicted increased identity disclosure, which was associated with *lower* levels of depression. In addition, participating in the community counterproductively predicted *higher* levels of depression via more frequent exposure to victimization.

These findings align with previous research demonstrating the positive associations of community participation with identity disclosure [22, 23] and victimization [25]. The results are also compatible with those of Elmer and colleagues [26], who reported that identity disclosure and victimization mediate the association of community participation with mental health outcomes among sexual minority adults. The findings suggest that community participation encourages SGM individuals to disclose their identities to more people and, therefore, is associated with lower depressive symptoms. Still, community participation is likely to be counterproductive through increased exposure to victimization, suggesting that individuals who participate in the community are expected to pay the price. Therefore, community participation can be a double-edged sword requiring SGM individuals to constantly evaluate the pros and cons and engage in a decision-making process [47, 48].

Moreover, for sexual minority men and women, it was found that the extent to which community participation indirectly predicted depression depended on structural stigma. Results revealed that the indirect effect of community participation on depression through increased identity disclosure was larger in more stigmatizing countries compared to less stigmatizing ones, suggesting that in more stigmatizing contexts, individuals enjoy the benefits of community participation more by disclosing their identities to more people. On the contrary, findings also indicated that community participation predicted higher levels of depression through elevated exposure to victimization among sexual minority men, with an amplified effect for those in high-stigma countries.

This result could be related to the intersectionality of sexual orientation and gender identity. Because being or being perceived as a cisgender heterosexual man has a privileged position in the society [49], sexual minority men who participate in the community and, therefore, are publicly open about their sexual identities to some extent, seem to lose their assumed heterosexually privileged position, especially in high-stigma countries, where more traditional gender norms and gender inequality exist [30]. Thus, it seems possible that sexual minority men in such countries pay the highest price for giving up or losing their assumed privileged position. Structural stigma being a nonsignificant moderator of community participation–victimization association for sexual minority women and gender minorities seems to support this argument, since they already do not have an assumed privileged position in the society to lose [49]. Similarly, studies comparing sexual minority men and women have consistently found that sexual minority men are exposed to victimization more frequently than sexual minority women [50]. Because masculine gender roles are less flexible than feminine ones, any perceived gender-atypical behavior of sexual minority women might go unnoticed or even sometimes welcomed [51], resulting in less exposure to victimization, unlike sexual minority men.

Furthermore, for gender minorities, structural stigma did not moderate the associations of community participation with either identity disclosure or victimization. This could be explained by the fact that gender identities are less concealable and more visible than sexual identities [31], which, therefore, might be associated with elevated identity disclosure and exposure to victimization regardless of structural stigma. Supporting this argument, previous studies also reported that while interpersonal stigma was positively associated with structural stigma for sexual minorities [11], the association was nonsignificant for gender minorities [10]. Subsample size differences might be another explanation, because our gender minority subsample was three to four times smaller than the subsamples of sexual minority women and men.

Nonetheless, it is also important to note that while the direct effect of community participation on depression was positive for sexual minorities, for gender minorities, it was negative, suggesting that community participation is directly and distinctively beneficial for gender minorities. Since many gender minorities, compared to sexual minorities, have more practical concerns, such as access to gender affirmative healthcare and legal procedures [31], this higher reliance on systemic structures might explain the negative direct effect of community participation on depression, as well as the invariability of community participation’s indirect effects on depression across countries. Still, those speculations could only be answered by further inquiry.

The current study’s findings provide important insights for interventions at individual, interpersonal, and structural levels. At the individual level, SGM individuals could be encouraged to participate in their communities, so that they will be more likely to disclose their identities, which has a positive impact on mental health. While doing so, however, it seems essential to consider the interpersonal consequences of community participation, such as exposure to victimization, which have detrimental impacts on mental health. Thus, individuals should be informed about the potential drawbacks of community participation and facilitated during their decision-making processes regarding the benefits and costs of community participation [48]. Yet, contextual factors such as structural stigma and the intersectionality of sexual orientation and gender identity should also be considered before applying interventions to increase community participation. Sexual minority men in high-stigma countries should especially be informed about the potential negative consequences of community participation resulting from the assumed privilege dynamics surrounding their identities. The findings also demonstrate the importance of changing legislation and social policies and reducing the bias in public attitudes regarding sexual orientation and gender identity to improve the well-being of SGM individuals.

Despite providing new insights, the current study has several limitations. First, the study used a cross-sectional design, preventing us from inferring causal or temporal relations between the variables. Thus, it is still not known whether community participation temporally antecedes or follows identity disclosure and victimization. Future studies using longitudinal designs are needed to shed light on the cause–effect relations. Another limitation is that the questions measuring study variables have not been widely validated, which might have limited the apprehension of the experiences of SGM individuals and attenuated our results. Therefore, future studies should validate these measures to better capture SGM individuals’ experiences. Furthermore, despite using data from a large sample of SGM individuals, since the subsamples were not necessarily representative of their respective countries, the generalizability of the results constitutes another limitation. More heterogeneous samples are needed in future studies to understand to what extent these findings could be generalized. In addition, the current study assessed behavioral participation in the SGM communities. However, cognitive and affective aspects of community involvement are also closely related to the mental health outcomes of SGM individuals [13, 14, 52]. Therefore, how cognitive and affective aspects of community involvement are associated with individual, interpersonal, and structural factors constitute another vital avenue for further investigation.

## Conclusions

The current study demonstrates, for the first time, that community participation is distinctively associated with depression among SGM individuals living in 28 European countries through higher levels of identity disclosure and victimization and as a function of the structural climates of the countries. It can be a double-edged sword, especially for sexual minority men in higher stigma countries who benefit from but at the same time are hindered by participating in the community the most. Thus, community-level interventions should consider the contextual factors and the intersectionality of sexual orientation and gender identity.

## Data Availability

Data are available under special license agreement with GESIS (https://www.gesis.org/en/home).
